# Smart Prussian Blue Analog Decorated with Zinc Oxide Nanohybrid: Fluorescent Sensing and Sustainability of Sunset Yellow in Food and Environment

**DOI:** 10.3390/bios15040263

**Published:** 2025-04-20

**Authors:** Hany A. Batakoushy, Amr K. A. Bass, Hassanien Gomaa, Sami El Deeb, Adel Ehab Ibrahim

**Affiliations:** 1 Department of Pharmaceutical Analytical Chemistry, Faculty of Pharmacy, Menoufia University, Shebin Elkom 32511, Egypt; 2 Department of Pharmaceutical Analytical Chemistry, Faculty of Pharmacy, Menoufia National University, 70 km Cairo-Alexandria Agricultural Road, Menoufia 32952, Egypt; amrk.a.bass@phrm.menofia.edu.eg; 3 Department of Pharmaceutical Chemistry, Faculty of Pharmacy, Menoufia University, Shebin Elkom 32511, Egypt; 4 Department of Chemistry, Faculty of Science, Al-Azhar University, Assiut 71524, Egypt; h.gomaa@azhar.edu.eg; 5 Institute of Medicinal and Pharmaceutical Chemistry, Technische Universitaet Braunschweig, 38106 Braunschweig, Germany; 6 Natural and Medical Sciences Research Center, University of Nizwa, P.O. Box 33, Birkat Al Mauz, Nizwa 616, Oman; 7 Pharmaceutical Analytical Chemistry Department, Faculty of Pharmacy, Port Said University, Port Said 42511, Egypt

**Keywords:** co-precipitation, PBA@ZnO, nanosheets, sunset yellow, fluorescence, food colorants

## Abstract

In the current study, the Prussian blue analog decorated with zinc oxide (PBA@ZnO) was produced using a simple chemical co-precipitation method. The nanohybrid was examined using XRD, EDX, SEM, and TEM techniques, where it exhibited a polycrystalline structure with highly intense broadening peaks. The surface morphology was observed as thin nanosheets decorated with tiny spheres. Following excitation at 360 nm, the fluorescence spectra of PBA@ZnO showed fluorescence emission at 455 nm. The developed PBA@ZnO was used to qualitatively and quantitatively assess sunset yellow (SY), where its native fluorescence was selectively quenched as SY concentrations increased. For the first time, PBA@ZnO was used as a turn-off nano-sensor for the spectrofluorimetric measurement of SY. The method’s markable sensitivity was demonstrated within an SY linearity range of 50–500 ng/mL, where the limit of detection was calculated as 9.77 ng/mL. Real sample analysis in the food industry, including samples from real food, soft drinks, and sun cream, was made possible by the detection of tiny amounts of SY. Analytical Greenness (AGREE), AGREEprep, and the complementing Green Analytical Procedure Index (Complex MoGAPI) were used to illustrate the new approach’s exceptional eco-friendliness and greenness. The RGB 12 algorithm worked to demonstrate that the suggested approach is less costly, more environmentally friendly, more sustainable, analytically sound, and whiter than the ones that were previously published. In accordance with ICH principles, the suggested method was validated. This approach offers a promising way to rapidly and accurately identify and measure SY in the food industry, helping to guarantee food safety and maintain the health of customers.

## 1. Introduction

The increasing use of synthetic dyes in the food and beverage industry has raised significant environmental and health concerns [1,2]. Sunset yellow (SY), a commonly used azo dye, is a notable example due to its potential toxicity and adverse effects on human health [3]. Its widespread application necessitates efficient and reliable methods for its detection and removal from environmental and food matrices. In this context, the development of advanced materials with multifunctional capabilities has become a priority for researchers [4,5].

Current analytical techniques for detecting SY, such as spectrofluorimetry [6,7], HPLC-UV [8,9], HPLC-MS [10,11], UV-spectrophotometry [12], and voltammetric methods [13,14] are highly accurate. However, they often require expensive equipment, skilled operators, and are time-consuming, especially during sample preparation steps. On the other hand, conventional remediation methods, including adsorption using activated carbon and chemical degradation, suffer from limited efficiency, high operational costs, and secondary pollution concerns. These limitations highlight the urgency of developing multifunctional materials capable of addressing both detection and remediation in an integrated and cost-effective manner.

Prussian blue analogs (PBAs) are a class of coordination compounds that are structurally related to Prussian blue (Fe_4_[Fe(CN)_6_]_3_), a deep blue pigment historically used in art and various applications. Fuel cells, pharmaceuticals, and energy storage devices all make extensive use of PBAs, where they have a wide range of applications in material science, electrochemistry, catalysis, and environmental science, owing to their small particle size, redox chemistry, high charge transfer, flexible molecular structure, and photomagnetic properties [15,16].

A simple chemical-based co-precipitation method was used to produce a Prussian blue analog decorated with zinc oxide (PBA@ZnO). The novel PBA@ZnO was characterized using different characterization tools, such as XRD, EDX, SEM, and TEM techniques. The prepared nanohybrid exhibited a polycrystalline structure with highly intense broadening peaks.

The whiteness meter was used to establish the level of the method’s sustainability, where it provides a simple evaluation tool for the proposed technique by applying the 12 White Analytical Chemistry (WAC) principles [17,18,19]. An alternative is to use (RGB 12) algorithms to select approaches that allow for a global assessment that considers all required criteria, such as those expressing respect for the WAC principles. The above-mentioned tools are therefore considered the best ones since they receive the greatest rating in this overall evaluation. An overview of the methods currently available for global whiteness evaluations of analytical processes is provided in a recently published work. For example, the recently created red, green, blue (RGB) model [9,20,21], refers to the color model commonly used in electronics and extends the concept of green chemistry to other basic colors (red and blue). Green indicates adherence to the principles of Green Analytical Chemistry (GAC); blue indicates practical/economic efficiency and productivity; and red indicates analytical efficiency as determined by validation criteria (accuracy, precision, LOD, etc.). Following review, the technique is assigned to a color that is determined from the proportion of each main color. This strategy was used to examine the created method’s sustainability and environmental friendliness, demonstrating its superior ecological impact. Additionally, the Analytical Greenness (AGREE) and supplementary Green Analytical Procedure Index (ComplexGAPI) were used to demonstrate the new approach’s exceptional eco-friendliness and greenness.

This study focuses on designing and applying Prussian blue analog decorated with zinc oxide (PBA@ZnO) nanosheets that combine fluorescence sensing and dye-removal capabilities in a single platform. The fluorescence-based detection mechanism offers high sensitivity and selectivity, while the nanosheets’ adsorption and catalytic features enable efficient dye removal from complex matrices. The proposed approach not only addresses the urgent need for real-time monitoring of food and environmental contaminants, but it also contributes to sustainable remediation practices. This work paves the way for developing advanced materials for integrated sensing and environmental applications.

The dual challenge of detecting and removing SY from food products and environmental matrices underscores the need for advanced, efficient, and sustainable solutions.

The possibility of generating a PBA@ZnO nanohybrid is the aim of this study. Variable tools were used for their characterizations, such as XRD, EDX, SEM, and TEM techniques, to explore their physicochemical properties. The prepared nanohybrid exhibited a polycrystalline structure with highly intense broadening peaks. Finally, the refined structure was used to reliably identify sunset yellow in samples of real food, soft drinks, and sunscreen. In order to improve food safety and consumer health, the established method established a promising sustainable approach for the quick and accurate detection and assessment of sunset yellow in the food industry.

## 2. Materials and Methods

### 2.1. Materials and Reagents

The German company Sigma-Aldrich provided the sunset yellow (>99% purity). All of the chemicals used were of analytical grades and did not require any additional processing. Zinc chloride dihydrate, potassium teteracyanonickelate [K_2_Ni(CN)_4_], potassium hexacyanocobaltate K_3_[Co(CN)_6_], zinc oxide, and Dimethyl Formamide (DMF) were obtained from Sigma-Aldrich (St. Louis, MO, USA) with purity (>99.9). The Aquatron Automatic Water Still A4000 (Staffordshire, UK) produced ultrapure water. To generate a variety of Britton–Robinson (BR) buffer solutions with pH ranges of 2.0–12.0, proportions of 0.04 M boric acid, phosphoric acid, and acetic acid solutions were mixed, and titrated to the desired pH using 0.2 M sodium hydroxide.

### 2.2. Preparation of PBA@ZnO Nanohybrid

The PBA (ZnTCNi/HCCo) was first prepared using the chemical co-precipitation method. Firstly, separate solutions of 0.2 moles of zinc chloride dihydrate (ZnCl_2_.2H_2_O), 0.12 moles potassium teteracyanonickelate [K_2_Ni(CN)_4_], and 0.05 moles potassium hexacyanocobaltate K_3_[Co(CN)_6_] were prepared in double distilled water using a magnetic stirrer, which were designated as solutions A, B, and C, respectively. Then, solution C was mixed with solution B and stirred for 3 h using a magnetic stirrer. Subsequently, solution A was gradually added to the combination above while being constantly stirred for 12 h. Centrifugation was then utilized to collect the precipitate, which was then repeatedly washed with double-distilled water and finally dried in an oven for 12 h at 75 °C. The nanohybrid ZnTCNi/HCCo@ZnO was made by partially dissolving 0.08 g of ZnO in 15 mL of deionized water, followed by mixing with PBA solution (0.5 g in 25 mL DMF) and stirring for 5 h using a magnetic stirrer. The mixture was then placed in a microwave oven for 40 min. The resulting precipitation followed washing, centrifugation, overnight drying at 80 °C, and 5 h of annealing at 300 °C.

### 2.3. Standard Sunset Yellow Solution

Sunset yellow (SY) stock solution (1 mg/mL) was made by dissolving 5.0 milligrams of SY in 1.0 mL ethanol in a 5 mL volumetric flask. After that, it was diluted with distilled water to the proper volume and kept refrigerated (at 4 °C) in dark amber containers. To establish the working solutions, the stock solution was further diluted with distilled water to provide a solution with a concentration of 100.0 µg/mL.

### 2.4. Real Sample Preparation

Food products like soft drinks and snacks (Doritos) were bought from the local market and consumed untreated. In a 50 mL measuring flask, 10 mL of the drink was diluted with distilled water to the appropriate level. Following the suggested protocol, a precise volume (1.0 mL) of this solution was handled. Blank samples of cosmetics, soft beverages, and Doritos snacks were examined for any traces of SY before the samples were spiked with known concentrations of SY. The calibration curve (50–500 ng/mL) was then used to determine the precise SY concentration in the original sample.

### 2.5. Sunblock Skincare Cream

In a volumetric flask, 1.0 g of SY (Photoderm^®^) cream was dissolved in 50 mL of methanol, heated for 5 min at 60 °C, and shaken continuously for three minutes. A final working concentration of 100 μg/mL of SY was obtained by filtering on a 100 mL volumetric flask and adding methanol until the mark was reached. The amount of SY in the original sample was ascertained by applying the suggested techniques to the examination of the dye under study in prepared solutions using the most appropriate possible procedure.

### 2.6. Calibration Graph Procedure

By dilution method, a volume of 1.5 mL of Britton–Robinson BR buffer (pH 3) was mixed with 0.5 mL of PBA@ZnO nanohybrid (5.0 mg/mL). The mixture was then placed in a 10 mL volumetric flask and mixed with 1.0 mL of SY working solution. The flasks were filled to the brim with ethanol to produce the final concentration range of 50–500 ng/mL. After 5 min, the fluorescence intensity was measured at λ-emission 455 nm (λ_ex_ 360 nm). The resulting fluorescence intensities were then plotted against the SY concentrations in ng/mL to construct the calibration curve.

### 2.7. Instrumentation

For the excitation and emission fluorimetric measurements, FS5 spectrofluorimeter equipment from Edinburgh instruments (Edinburgh, UK) was utilized, which was fitted with a 150 W xenon light source, a 1 cm quartz cell, and Fluoracle^®^ software (v.2.13.2, Edinburgh Instrument, Edinburgh, UK). The slit widths were set at 2 nm, and the instrument’s speed was 1000 nm/min. UV/Vis absorption spectra were acquired using Jasco V-570 spectrophotometer (Tokyo, Japan) using a 1.0 cm path-length quartz cell. The crystal structure of the prepared nanohybrid was investigated using a conventional Bruker X-ray diffraction (XRD), D8-VENTURE from Bruker (Billerica, MA, USA) with Cu Kα radiation, λ = 1.5406 Å at 2θ angle range of 15–60°. Energy-dispersive X-ray analysis (EDX) was employed to determine the elemental composition and purity of the sample. The surface morphology, shape, and internal structure of the nanoparticles were visualized by scanning electron microscopy (SEM; FEI Helios Nanolab 400). The transmission electron microscopy (TEM) was acquired by Jeol JEM-1400 from JEOL Ltd. (Tokyo, Japan), operating at an accelerating voltage of 100 kV to determine the developed PBA@ZnO nanohybrid’s shape, dimensions, and particle’s size.

## 3. Results

### 3.1. Structural and Morphological Analysis

**Figure 1:**
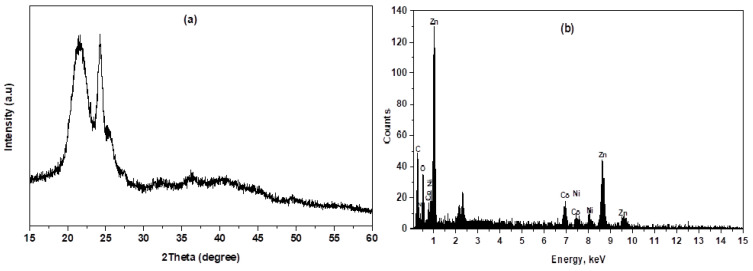
(**a**) XRD pattern and (**b**) EDX spectrum of PBA@ZnO nanohybrid.

The XRD pattern of Prussian blue analog-doped zinc oxide ZnTCNi/HCCo@ZnO annealed at 300 °C for 5 h is depicted in Figure 1. The sample exhibited a polycrystalline structure with diffraction peaks observed at 2θ = 21.75°, 24.30°, 32.15°, 35.80°, and 49.80° corresponding to the reflection planes (200), (220), (400), (420), and (600), respectively, which is matched with the face-centered cubic phase of the standard card (JCPDS card No. 01–0239) and similar to the XRD pattern in the literature [22,23,24]. As shown in Figure 1a, the sample had a relatively good crystalline structure; however, the broadening peaks indicate a small crystalline size. No peaks ascribed to impurities or starting unreacted ions were observed in the pattern, suggesting the purity of the nanocomposite. The XRD parameters, including crystallite size (D), lattice strain (ε), dislocation density (δ), and degree of crystallinity (X_c_), were identified from the reflection peaks (200) and (220) using the following relationships: $D=\frac{k\lambda }{\beta cos\theta }$ (Scherrer equation), $\varepsilon =\frac{\beta }{4tan\theta }$ (Stokes-Wilson formula), $\delta =\frac{1}{D^{2}}$, and $X_{c}=\frac{0.24}{\beta }$, respectively, where, in these equations, β is the full width at half maximum (FWHM) of the corresponding peaks measured in radians, k represents the shape factor equals 0.94, θ is the diffraction angle, and λ denotes the wavelength of the incident X-ray = 1.541 Å for Cu Kα radiations [25,26]. The crystallite size was calculated to be 23.70 nm; moreover, a large value of internal strain may be attributed to the vicinity of non-uniform crystal lattice distortion and a weak crystallinity. The crystallography parameters of the nanohybrid were summarized in Table 1, indicating considerable internal strain and dislocation density [15].

**Figure 2:**
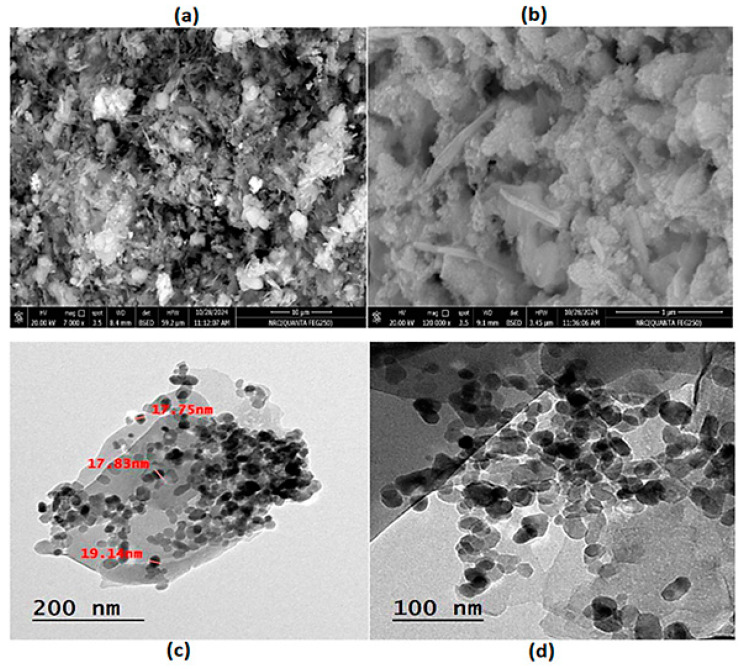
(**a**,**b**) SEM images and (**c**,**d**) TEM images of PBA@ZnO nanohybrid.

The chemical composition, purity, and element weight percentage (Wt%) of the nanohybrid were verified using the EDX spectrum. EDX is a powerful analytical technique used to characterize the elemental composition of materials. The EDX spectrum displays peaks corresponding to different elements, that confirm which elements are present in the nanohybrid. Figure 1b shows the detected elements C, N, Zn, O, Co, and Ni, with Wt% of 2.78, 13.42, 37.60, 11.79, and 34.41%, respectively. The results of the EDX confirmed the formation of a ZnTCNi/HCCo@ZnO nanocomposite, as previously demonstrated by Elgazzar in his reported Prussian blue analog [24]. Moreover, the EDX analysis confirms the formation of the nanohybrid by showing Zn and O in the spectrum, indicating the successful doping of ZnO into the host PBA framework with Wt% coinciding with the quantitative measure of the doping concentration as previously demonstrated by Mostafa et al. [27] and Hazem et al. [28]. The spectrum also revealed that the approximate stoichiometry of the nanohybrid composition contains Zn, Co, Ni, C, and N, confirming their successful doping in the hybrid nanocomposite. The SEM and TEM spectroscopy were examined at different magnifications to visualize size, shape, dimensions, and surface nature. The SEM images show the surface of the nanohybrid in flat sheet-like structure; however, little nanoparticles appeared in a cube shape, and some of them took a round shape, as can be seen in Figure 2a [29,30].

At high magnification, as depicted in Figure 2b, the nanosheets were non-uniformly distributed, slightly rough, with cracks due to the aggregation and large particle density. Furthermore, in the TEM and SEM images, the nanosheets are thin and exhibit an average size of approximately 19 nm with a typical size about 25 nm. The surface of the nanosheets is decorated with tiny nanospheres [31]. As shown in Figure 2c,d, the nanosheets are flat with a large specific surface area and large aspect ratio, meaning their length is much larger than their thickness [32,33]. Some of the nanoparticles possess hexagonal shapes, which are attributed to the presence of ZnO in the matrix. Padhan et al. [34] studied their synthesized ZnO nanoparticles using TEM that showed a hexagonal crystal structure, and then this was confirmed by the EDX spectral analysis. ZnO nanostructures developed by Moumen et al. also showed hexagonal morphology, which was confirmed by EDX analysis of their developed nanorods [35]. Thus, in the proposed study, the hexagonal-shaped morphology of some of the nanoparticles as seen in TEM images (Figure 2c,d), together with their analyzed EDX spectrum (Figure 1b), confirmed the incorporation of ZnO into the introduced nanohybrid.

### 3.2. Fluorescence Quantum Yield of (*PBA@ZnO*) Nanohybrid

Using quinine sulfate as the control, the single-point technique was used to investigate the fluorescence quantum yield (QY) of the PBA@ZnO nanohybrid [36]. Quinine sulfate (QS) has a quantum yield of 54% and a refractive index (η_std_) of 1.33 in a 0.1 M H_2_SO_4_ solution [37]. The QY of the PBA@ZnO nanohybrid was determined using the following formula:

(1) $$Q_{PBA@ZnO}=Q_{Quinine}\times \frac{F_{PBA@ZnO}}{F_{Quinine}}\times \frac{A_{st}}{A_{PBA@ZnO}}\times \frac{\eta^{2}PBA@ZnO}{\eta^{2}Quinine}$$

The suggested method generates more optical effects on the surface of PBA@ZnO, resulting in a high quantum yield. The quantum yield of PBA@ZnO was calculated to be as high as 38.1% according to Equation (1), where Q stands for quantum yield, F represents the integrated fluorescence, A is the absorbance, and η stands for the solvent refractive index.

### 3.3. Optical Characters of the Synthesized *PBA@ZnO* Nanohybrid

The UV spectrum of PBA@ZnO exhibits a maximum absorption peak at 270 nm, as shown in Figure S1. The peak matches the n-π* electronic transition of the PBA@ZnO nanohybrid’s surface. When exposed to UV light, the generated PBA@ZnO nanohybrid showed intense blue fluorescence and a yellow color under optical light.

**Figure 3:**
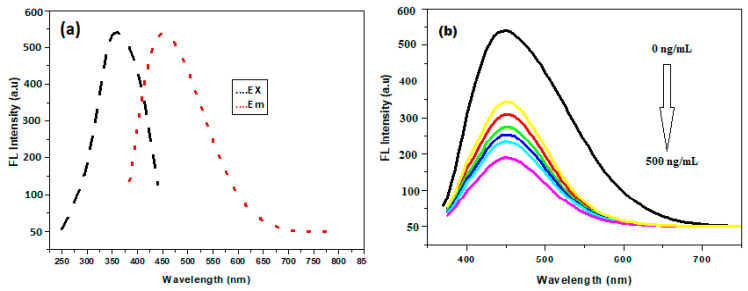
(**a**) Excitation and emission spectra of PBA@ZnO nanohybrid (at 360 and 455, respectively); and (**b**) Fluorescence quenching using 0.5 mL of PBA@ZnO nanohybrid at pH 3, 1.5 mL of BR buffer, and the complete response was determined after 5 min.

The excitation-dependent emission behavior of PBA@ZnO was investigated over the excitation wavelength range of 330–380 nm. As the excitation wavelength increased, the relative fluorescence intensity (RFI) peaked between 330 and 360 nm, and the RFI decreased again when leaving the 360–380 nm range (Figure S2a). This in turn confirms the excitation-dependent nature for the PBA@ZnO spectra [38]. The differences in fluorescence maxima of the nanohybrids as a function of excitation wavelength could arise from one or a combination of several factors such as size effects, electronic transitions, and/or the inherent properties of the nanohybrid materials. The optimal excitation wavelength, which produces the maximum excitation intensity, was determined to be 360 nm with fluorescence emission maximum at 455 nm, as shown in Figure 3a. Digital images of PBA@ZnO nanoprobes showing their strong fluorescence are shown in Figure S2b, also showing their quenching upon addition of SY (Figure S2b) under UV excitation and under white light (Figure S2c).

### 3.4. Optimization of the Developed Method

**Figure 4:**
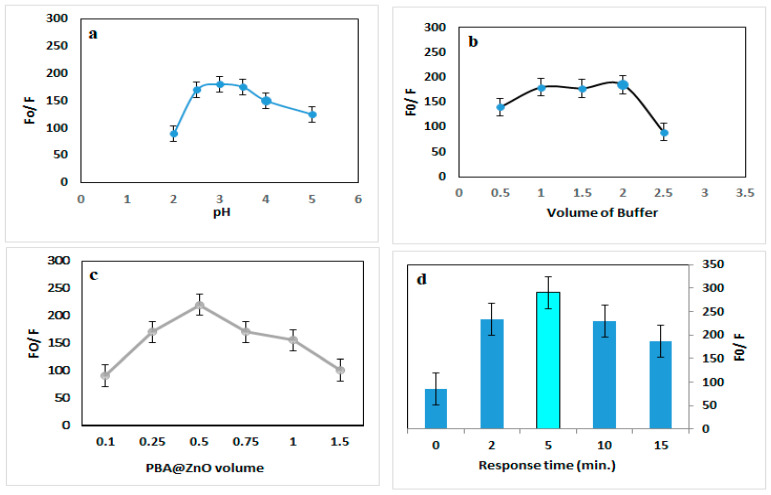
Effect of (**a**) pH, (**b**) buffer volume, (**c**) PBA@ZnO volume, and (**d**) response time on the quenching of PBA@ZnO fluorescence by SY (200 ng/mL).

The native fluorescence of PBA@ZnO was gradually quenched by the addition of SY as the concentrations increased (Figure 3b). The sensitivity of the proposed method to the SY was enhanced by adjusting the pH, buffer volume, PBA@ZnO volume, and response time. The effect of a range of pH values from 2.0 to 5.0 on PBA@ZnO’s fluorescence response with SY was investigated. Furthermore, the buffer volume was examined within the 0.5–2.5 mL range. The best quenching effect was achieved at pH 3 and 1.5 mL of BR buffer, as illustrated in Figure 4a,b.

Using varying volumes (0.1–1.5 mL), the effect of PBA@ZnO volume on the fluorescence response with SY was investigated. The most stable quenching was obtained with 0.5 mL volume of PBA@ZnO, as shown in Figure 4c. Various incubation times (0–20 min) were examined in order to identify the ideal time for maximal fluorescence quenching. Partial quenching of fluorescence was seen immediately after mixing (0 min), suggesting an early interaction. Figure 4d illustrates that fluorescence reached its peak at an optimal response time of 5 min, followed by a subsequent decline (Figure 4d). This rapid response is advantageous for real-time and on-site detection applications. Meanwhile, the slight decline in fluorescence sensitivity can be understood by understanding the interaction mechanism. As would be explained in the next subsection, the interaction between SY and PBA@ZnO was attributed to dynamic quenching. In dynamic quenching, the quenching occurs due to collisional interactions between the nanohybrid and the analytes. As time progresses, the frequency of such collisions may decrease, leading to reduced quenching [39]. While decreasing quenching efficiency over time can be a limitation, optimizing interaction conditions and leveraging kinetic analysis can turn this into an advantage for sensitive, selective, and real-time detection, as proven by the optimization and control of the other factors.

### 3.5. Suggested Interreaction Mechanism of the Proposed Method

As shown in Figure 3, the fluorescence intensity of the produced PBA@ZnO nanohybrid was quantitatively quenched by the addition of increasing quantities of SY. PBA@ZnO could interact with other elements through a variety of methods, including the inner filter effect, dynamic quenching, and static quenching [15,40].

The excitation/emission spectra of PBA@ZnO at 360/455 nm, respectively, do not overlap with the UV spectrum of SY (λ = 259 nm). The inner filter effect mechanism was so disregarded. Therefore, the Stern–Volmer equation was used to differentiate between static and dynamic quenching:

$$F/Fo=1+K_{sv}[Q]$$

where [Q] is the SY molar concentration, K_SV_ is the Stern–Volmer quenching constant, and (F_0_) and (F) are the fluorescence intensities of PBA@ZnO in the presence and absence of the investigated dye, respectively.

The suggested method was used at various temperatures (293, 303, and 313 K) to examine the quenching mechanisms. The K_SV_ constant grows with temperature in dynamic quenching but decreases with temperature in static quenching. Temperature has no effect on the K_SV_ constant in the case of inner filter effect [41]. The inner filter effect is a phenomenon that occurs in fluorescence spectroscopy, leading to inaccuracies in the quantification of fluorescence signals. It arises when the excitation or emission light is absorbed by the sample itself or by other components in the solution, affecting the intensity of the detected fluorescence [42]. The dynamic quenching mechanism was shown by the calculated K_SV_ values, which were 0.0067, 0.0071, and 0.0079 at 293, 303, and 313 K, respectively (Figure S3b). Moreover, because PBA@ZnO and SY have several functional groups, the interaction may be explained by the electrostatic attraction between them.

### 3.6. Method’s Validation

The suggested method was verified using the International Conference on Harmonization’s (ICH) guidelines [43]. The calibration graph was created by plotting F_0_/F against SY concentrations (ng/mL) (Figure 3b and Figure S3a). It was determined that the calibration range was between 50 and 500 ng/mL, as shown in Table 2. With a correlation value of 0.9998, the regression formula was found to be y = 0.9616x + 140.58.

The following formulas were used to calculate the detection (LOD) and quantitation limits (LOQ): LOQ = 10 _6_/slope, LOD = 3.3 _6_/slope, where ϭ is the intercept’s standard deviation. With LOD and LOQ of 9.77 and 29.62 µg/mL, respectively, as shown in Table 2, the suggested method’s high sensitivity made it possible to analyze SY in real samples.

In triplicate, five concentrations (50, 100, 200, 300, and 400 ng/mL) were used to test the accuracy of the suggested method. The method’s great accuracy was demonstrated by recovery results, which ranged from 98.97% to 101.82% with relative standard deviation (RSD) values between 0.44 and 1.20 (Table 3).

Using three concentration levels (100, 200, and 300 ng/mL) at three replicate measurements on the same day and over three consecutive days, respectively, the intra-day and inter-day precisions were investigated. As seen by the low percentage RSD values obtained (≤0.55), the results demonstrate high precision for the suggested approach (Table 3). The method’s suitability and reliability for the sensitive and selective determination of SY are demonstrated by the high recovery percentage values (98.74–100.21).

To assess the interference from various sources, the suggested method’s selectivity was investigated. Various food coloring agents, including Allura Red and Indigo Carmine, were used to examine the suggested method’s selectivity. The competitive detection study tested whether PBA@ZnO sensor can selectively detect SY in the presence of other similar substances’ “competing analytes” and the fluorescence titration with other possible interferences (as showed in Figure S5). Even when different dyes are present, the results demonstrate the good selectivity of the suggested approach for determining SY. The excess concentration of 1.0 µg/mL, which is almost ten times greater than the used SY concentration (100 ng/mL), was applied to the tested interferences. They did not considerably reduce the PBA@ZnO’s fluorescence, demonstrating the high selectivity of the recommended strategy. Additionally, because the fluorescence of the PBA@ZnO was not quenched by the presence of certain vitamins found in orange juice, like vitamin C, niacin, and thiamin (1.0 µg/mL), the method was able to detect SY without interference; similarly, the method’s extraordinary selectivity was confirmed by its ability to detect SY without interference when metal ions, such as sodium, potassium, calcium, and magnesium (1.0 µg/mL), were present (Figure S4).

### 3.7. Real Sample Determination of SY

The proposed method was applied to identify SY in soft drinks and food coloring. Blank samples of cosmetics, soft beverages, and Doritos snacks were examined for any traces of SY before the samples were spiked with known concentrations of SY (100, 200, and 300 ng/mL). Prior to the spiking tests, the examined samples did not contain detectable amounts of the dye, as evidenced by an absence of substantial fluorescence or absorbance signals corresponding to SY. After spiking, the recovery percentage values were calculated as indicated in Table 4. The results indicate the precision of the recovery investigations and the reliability of the method in identifying SY in actual samples. The method’s appropriateness and reliability for the sensitive and selective determination of SY are demonstrated by the high recovery percentage values (99.77–100.94%) that were obtained.

### 3.8. Comparison Study of the Proposed and the Reported Methods for the Determination of SY

The developed PBA@ZnO nanohybrid-based probe was contrasted with other reported techniques that had already been published (Table 5). Compared to the reported HPLC-UV technique using gradient elution [8], and the reported technique using solid phase extraction [44], the proposed tools showed much higher sensitivity, as indicated by the LODs, which need extensive sample preparation, large quantities of organic solvents, and complicated equipment. Meanwhile, the proposed tool exhibited almost the same sensitivity explored by the LC-MS technique [11]. However, the use of the PBA@ZnO nanohybrid offers much easier to use, quick, economical, and ecologically friendly bases. Although LC-MS is a powerful analytical technique widely used in various fields, the high equipment and maintenance costs, together with the technical complexity that requires expert operation, are major disadvantages. Consequently, the novel technology offered a spectrofluorimetric methodology for SY measurement that is ultra-sensitive, quick, cost-effective, time-saving, and straightforward employing a PBA@ZnO nanohybrid with a high degree of greenness. Additionally, the established approach was used to determine SY in sunscreen cream, soft beverages, and food coloring that most of the developed technologies were unable to complete. For determining SY, the developed probe is therefore better than the ones that have been previously published.

### 3.9. Evaluations of Sustainability

#### 3.9.1. Whiteness Metric

The red, green, blue (RGB 12) tool [18], created by Pawe-Nowak and colleagues in June 2021, provides a straightforward evaluation of WAC principles. The RGB 12 algorithm is a color-based method used for evaluating the greenness (environmental friendliness) of an analytical method by analyzing the red, green, and blue (RGB) components of a colored representation (pictogram) associated with the method. The algorithm assigns a score based on the dominance of the green component in the RGB color space, indicating how environmentally sustainable the method is. The twelve principles that comprise the RGB 12 score are divided into four primary groups in each category: reds, greens, and blues. Red principles encompass the analytical performance of the method in terms of sensitivity and validation. Green principles cover the green chemistry concepts regarding the use of reagents and the method’s impact on humans and the environment. Finally, blue principles cover the practicality and applicability of the method. The key features of GACs, such as the method’s used chemicals toxicity, waste production, energy consumption, and possible effects on people, animals, and genetically modified organisms (G1–G4), are the emphasis of the green subgroup. The red subgroup (R1-R4) focuses on validation measurements. The blue subgroup (B1-B4) is concerned with cost-effectiveness, time-efficiency, and economic objectives. The scores generated by the procedure for each of the three colored zones using the RGB algorithm are added to determine the sum value of “whiteness”, which assesses how well the methodology conforms with WAC criteria. As shown in Figure S6, the current approach is more economical, environmentally friendly, whiter, sustainable, and analytically efficient than the published ones [44].

#### 3.9.2. Greenness Assessment

To achieve a high level of greenness, green chemistry is focused on eliminating all risks associated with chemical processes. Analytical processes’ beneficial environmental effects are increasingly being evaluated, and several ways are being used to compare them [47]. Greenness assessment metrics, namely, AGREE [48], AGREEprep [49] and Complex MoGAPI [50], were used to analyze the developed approach’s eco-friendliness and greenness.

An enhanced version of GAPI called Complex MoGAPI evaluates the environmental friendliness of the analytical method it uses as well as the pre-processing steps. An extra hexagonal shape was added to the GAPI pictogram in Complex MoGAPI in order to assess the environmental friendliness of a number of factors, including solvents and reagents, workup and instruments, conditions, yield, and purification. AGREE is a quantitative technique that evaluates 12 GAC critical factors to determine the environmental and occupational risks of an analytical procedure. The numerical scale for AGREE goes from 0 to 1 [51]. A procedure is considered more environmentally friendly if it is closer to one. The sample preparation is given priority by the AGREEprep^®^ Calculator metric tool, which is based on ten environmental factors that are converted to sub-scores on a 0–1 scale before being utilized to determine the final assessment score. Solvents, materials and reagents, waste production, energy use, sample size, and throughput are all factors that affect each score.

The PBA@ZnO nanohybrid was synthesized using non-toxic ingredients in the suggested technique. Furthermore, BR buffer and ethanol were employed as green solvents for the direct measurement of sunset yellow. With a significant AGREE score of 0.77, AGREEprep score of 0.82, and green-colored Complex GAPI score of 86, as illustrated in Figure S7, it was determined that the approach is good green.

## 4. Conclusions

An innovative, swift, and eco-friendly spectrofluorimetric method for evaluating SY was devised in the present work. The method depends on generating an environmentally friendly PBA@ZnO nanohybrid. In response to UV light, the synthesized PBA@ZnO nanohybrid showed blue fluorescence. XRD, EDX, SEM, and TEM techniques were used to describe the PBA@ZnO structure and identify its characteristics. This technique is the first analytical study that describes the use of the PBA@ZnO nanohybrid to determine SY. SY quenched the produced nanohybrid’s intrinsic fluorescence, and the method’s validation showed acceptable percentage recoveries and percentage relative standard deviation. The suggested method successfully evaluated SY in real samples including food snacks, soft beverages, and sunscreen cream and showed outstanding sensitivity and selectivity. Considering the ecological footprint, the proposed procedure showed a high degree of greenness and whiteness. The developed technique presents a viable strategy for a high throughput and accurate identification and assessment of SY in the food industry, which should help enhancing consumer health and food safety.

## Figures and Tables

**Table 1:** XRD parameters of PBA@ZnO nanosheets.

Nanohybrid	D (nm)	$\delta \times 10^{-4}\;{\left(\mathrm{nm}\right)}^{-2}$	$\varepsilon \times 10^{-3}$	$X_{c}$
PBA@ZnO	23.70	17.80	6.79	17.25

**Table 2:** Fluorescence analytical performance parameters for determination of SY with PBA@ZnO nanohybrid.

Parameter	SY
Wavelength, λ_ex_/λ_em_ (nm)	360/455
Linearity range (ng/mL)	50–500
LOD (ng/mL)	9.77
LOQ (ng/mL)	29.32
Intercept ± SD_a_ (s_a_)	140.58 ± 2.84
Slope ± SD_a_ (s_b_)	0.9616 ± 0.009
Correlation coefficient (r)	0.9998
SD of residuals (S_y/x_)	3.66
(Intra-day precision) % Recovery ± RSD	(98.74–100.21) ± (0.55–1.15)
(Inter-day precision) % Recovery ± RSD	(99.15–99.98) ± (0.57–1.22)

**Table 3:** Accuracy and precision results for the intereaction of SY with PBA@ZnO nanohybrid.

NO.	Added Conc. (ng/mL)	Found Conc. (ng/mL)	% Recovery * ± RSD
1	50	50.91	101.82 ± 0.44
2	100	100.61	100.61 ± 0.67
3	200	197.94	98.97 ± 1.20
4	300	297.33	99.11 ± 1.07
5	400	405.02	101.25 ± 0.76
Intra-day precision	100	99.57	99.46 ± 0.55
	200	200.43	100.21 ± 0.68
	300	296.22	98.74 ± 1.15
Inter-day precision	100	99.98	99.98 ± 0.57
	200	198.50	99.25 ± 0.67
	300	297.45	99.15 ± 1.22

* Mean of three determinations. RSD: Relative standard deviation.

**Table 4:** Analysis of SY in different real samples using the PBA@ZnO nanohybrid.

Sample	Added Conc. (ng/mL)	Found Conc. (ng/mL)	% Recovery * ± RSD
Doritos Snaks	100	100.55	100.55 ± 1.20
	200	201.13	100.56 ± 0.67
	300	299.32	99.77 ± 1.09
Soft Drink	100	100.76	100.76 ± 0.79
	200	200.60	100.30 ± 0.90
	300	299.62	99.87 ± 1.42
Skincare Cream	100	100.94	100.94 ± 0.71
	200	200.67	100.34 ± 0.55
	300	299.61	99.98 ± 0.80

* Mean of six determinations.

**Table 5:** Comparison between the proposed and reported methods for SY analysis.

No.	Analytical Method	Linearity Range	LOD	Real Sample	Ref.
(1)	HPLC	1–20 µg/mL	70.0 ng/mL	Mineral water	[44]
(2)	HPLC	1–100 µg/mL	30.0 ng/mL	Soft Drinks	[8]
(3)	Chemometrics analysis	2–29 µg/mL	0.9 µg/mL	Soft Drinks	[45]
(4)	Fluorescence (CDs-PTD)	0–180	106.8 × 10^−9^ M	Beverages	[46]
(5)	HPLC-MS	1–1000 ng/mL	2.2 ng/mL	Animal feeds and meat	[11]
(6)	Fluorimetry: PBA@ZnO nanohybrid	50–500 ng/mL	9.8 ng/mL	Food snacks, soft drinks, sunscreen cream	The present Work

## Data Availability

The manuscript encompasses the whole information related to or associated with the research work.

## Supplementary Materials

The following supporting information can be downloaded at https://www.mdpi.com/article/10.3390/bios15040263/s1, Figure S1: UV spectrum of PBA@ZnO nanohybrid (5.0 mg/mL) showing peak maximum at 270 nm; Figure S2: Fluorescence emission spectra of PBA@ZnO (5.0 mg/mL) nanohybrid at different excitation wavelengths (330–380 nm), and digital images of non-spiked PBA@ZnO under UV and white light (b&c left cuvette) and PBA@ZnO spiked with SY under UV and white light (b&c right cuvette); Figure S3: (a) Calibration curve of increasing concentrations of SY (0, 50, 100, 200, 300, 400, 500 ng/mL) using 0.5 mL of PBA@ZnO nanohybrid at pH 3, 1.5 mL of BR buffer, and the complete response was determined after 5 min, and (b) Stern–Volmer plot for the quenching effect of PBA@ZnO nanohybrid by SY at different temperatures (293, 303, 313 K); Figure S4: Selectivity of the proposed PBA@ZnO nanohybrid (100 ng/mL) with SY alternatives (1.0 µg/mL); Figure S5: (a) Selectivity of the proposed PBA@ZnO nanohybrid (100 ng/mL) with competing analytes, (b) fluorescence titration with other analytes; Figure S6: Comparison of RGB 12 algorithm of the suggested approach to the reported HPLC method; Figure S7: Assessment of the greenness profile of the proposed method by (a) AGREE, (b) AGREE prep, and (c) Complex MoGAPI.

## References

[1] Sahraei R., Farmany A., Mortazavi S. (2013). A nanosilver-based spectrophotometry method for sensitive determination of tartrazine in food samples. Food Chem..

[2] Zhu J., Li C., Liu S., Liu Z., Yang J., Tian J., Hu X. (2014). A non-diazotization-coupling reaction-based colorimetric determination of nitrite in tap water and milk. Eur. Food Res. Technol..

[3] Jalali Sarvestani M.R. (2024). A Review on Sunset Yellow Toxicity and its Analytical Methods. J. Chem. Biol. Med. Sci..

[4] Mohiuddin A. (2019). The mysterious domination of food/Drinking water contaminants and adulterants in Bangladesh. PharmaTutor.

[5] Liang J. (2022). Nutritional Toxicology.

[6] Zhang Q., Wang X., Yuan L., Yu L., Shao C., Jia H., Lu S. (2024). Nitrogen-doped biomass-derived carbon dots for fluorescence determination of sunset yellow. Anal. Methods.

[7] Yuan Y., Zhao X., Qiao M., Zhu J., Liu S., Yang J., Hu X. (2016). Determination of sunset yellow in soft drinks based on fluorescence quenching of carbon dots. Spectrochim. Acta Part A Mol. Biomol. Spectrosc..

[8] Agbokponto J.E., Kpaibe A.P.S., Yemoa L.A., Assanhou A.G., Ganfon H., Gbassi G.K., Aké M. (2022). Simultaneous determination by HPLC-UV vis of tartrazine and sunset yellow in soft drinks sold in benin. Am. J. Anal. Chem..

[9] Chakraborty A., Jayaseelan K. (2024). Analytical Quality by Design aided RP-HPLC method for the estimation of Sunset Yellow in commercial food samples employing green ultrasound assisted extraction: Greenness, Blueness and Whiteness evaluation. Green Anal. Chem..

[10] Gosetti F., Gianotti V., Polati S., Gennaro M.C. (2005). HPLC-MS degradation study of E110 Sunset Yellow FCF in a commercial beverage. J. Chromatogr. A.

[11] Zou T., He P., Yasen A., Li Z. (2013). Determination of seven synthetic dyes in animal feeds and meat by high performance liquid chromatography with diode array and tandem mass detectors. Food Chem..

[12] Dinç E., Baydan E., Kanbur M., Onur F. (2002). Spectrophotometric multicomponent determination of sunset yellow, tartrazine and allura red in soft drink powder by double divisor-ratio spectra derivative, inverse least-squares and principal component regression methods. Talanta.

[13] Peña-Gonzalez A., García-Beltrán O., Nagles E. (2018). Detection of sunset yellow by adsorption voltammetry at glassy carbon electrode modified with chitosan. Int. J. Electrochem. Sci..

[14] Calam T.T., Çakıcı G.T. (2023). Optimization of square wave voltammetry parameters by response surface methodology for the determination of Sunset yellow using an electrochemical sensor based on Purpald^®^. Food Chem..

[15] El Mously D.A., Mahmoud A.M., Abdel-Raoof A.M., Elgazzar E. (2022). Synthesis of prussian blue analogue and its catalytic activity toward reduction of environmentally toxic nitroaromatic pollutants. ACS Omega.

[16] Naggar A.H., Seaf-Elnasr T.A., Thabet M., El-Monaem E.M.A., Chong K.F., Bakr Z.H., Alsohaimi I.H., Ali H.M., El-Nasser K.S., Gomaa H. (2023). A hybrid mesoporous composite of SnO_2_ and MgO for adsorption and photocatalytic degradation of anionic dye from a real industrial effluent water. Environ. Sci. Pollut. Res..

[17] Batakoushy H.A., Hafez H.M., Soliman M.M., Mohamed T.F., Ahmed A.B., El Hamd M.A. (2024). Isoquinoline-based intrinsic fluorescence assessment of erythropoiesis-stimulating agent, Roxadustat (FG-4592), in tablets: Applications to content uniformity and human plasma evaluation. Luminescence.

[18] Nowak P.M., Wietecha-Posłuszny R., Pawliszyn J. (2021). White Analytical Chemistry: An approach to reconcile the principles of Green Analytical Chemistry and functionality. TRAC Trends Anal. Chem..

[19] Abdel-Lateef M.A., Darwish I.A., Gomaa H., Katamesh N.S. (2024). Design of resonance Rayleigh scattering and spectrofluorimetric methods for the determination of the antihistaminic drug, hydroxyzine, based on its interaction with 2, 4, 5, 7-tetraiodofluorescein: Evaluation of analytical eco-scale and greenness/whiteness algorithms. Luminescence.

[20] Tobiszewski M., Marć M., Gałuszka A., Namieśnik J. (2015). Green chemistry metrics with special reference to green analytical chemistry. Molecules.

[21] Sajid M., Płotka-Wasylka J. (2022). Green analytical chemistry metrics: A review. Talanta.

[22] Xu L., Liu Y., Chen M., Wu W., Qiu S., Wu H., Zheng M., Zhang X., Wu X. (2025). Suppressing vacancies and crystal water of sodium manganese iron-based Prussian blue analogue by potassium doping for advanced sodium-ion batteries. Chem. Eng. Sci..

[23] Mosaad S., Ibrahim A.H., Elesh E., El-Damhogi D.G., Elgazzar E. (2023). Synthesis of prussian blue analog (Co/TCNi/HCCr) nanoparticles using a facile co-precipitation approach and evaluation of their dielectric characteristics for electronic applications. J. Mater. Sci. Mater. Electron..

[24] Elgazzar E. (2020). Prussian blue analogue cobalt tetracyanonickelate hexacyanochromate decorated by CNTs: Structural, morphological, optical characterization. Mater. Res. Express.

[25] Pal S., Jana S., Singh D.K., Ganesan V., Azad U.P., Prakash R. (2024). Engineered Ni–Fe prussian blue analogue nanocubes and their transformation into nanocages and mixed oxide for applications as bifunctional electrocatalyst. Int. J. Hydrogen Energy.

[26] Zhang H., Jiang Q., Hadden J.H., Xie F., Riley D.J. (2021). Pd ion-exchange and ammonia etching of a prussian blue analogue to produce a high-performance water-splitting catalyst. Adv. Funct. Mater..

[27] Mostafa W.A., Elgazzar E., Beall G.W., Rashed S.S., Rashad E.M. (2018). Insecticidal effect of zinc oxide and aluminum oxide nanoparticles synthesized by co-precipitation technique on Culex quinquefasciatus larvae (Diptera: Culicidae). Int. J. Appl. Res..

[28] Hezam F.A., Nur O., Mustafa M.A. (2020). Synthesis, structural, optical and magnetic properties of NiFe2O4/MWCNTs/ZnO hybrid nanocomposite for solar radiation driven photocatalytic degradation and magnetic separation. Colloids Surf. A Physicochem. Eng. Asp..

[29] Gu Y., Lu Y., Dai P., Cao X., Zhou Y., Tang Y., Fang Z., Wu P. (2025). Self-assembled high-entropy Prussian blue analogue nanosheets enabling efficient sodium storage. J. Colloid Interface Sci..

[30] Wang J., Wang L., Li Z., Xu L., Zhang L., Bao K., Li T., Chen L. (2024). Fe-doped phosphide nanosheet array derived from prussian blue analogues for high-efficient electrocatalytic water splitting. Int. J. Hydrogen Energy.

[31] An X., Quan L., Liu J., Tang Q., Lan H., Liu H. (2022). Mo, Fe-codoped metal phosphide nanosheets derived from Prussian blue analogues for efficient overall water splitting. J. Colloid Interface Sci..

[32] Yu M., Li Z., Shi H., Lin S., Zhang X., Mo F., Lai F., Liang D. (2022). Preparation of graphite carbon/Prussian blue analogue/palladium (GC/PBA/pd) synergistic-effect electrocatalyst with high activity for ethanol oxidation reaction. Int. J. Hydrogen Energy.

[33] Elshorbagy M., Ramadan R., Abdelhady K. (2017). Preparation and characterization of spray-deposited efficient Prussian blue electrochromic thin film. Optik.

[34] Padhan S., Wagri N.K., Dash L., Das A., Das S.R., Rafighi M., Sharma P. (2023). Investigation on Surface Integrity in Hard Turning of AISI 4140 Steel with SPPP-AlTiSiN Coated Carbide Insert under Nano-MQL. Lubricants.

[35] Moumen A., Kaur N., Poli N., Zappa D., Comini E. (2020). One Dimensional ZnO Nanostructures: Growth and Chemical Sensing Performances. Nanomaterials.

[36] Rurack K. (2008). Fluorescence quantum yields: Methods of determination and standards. Stand. Qual. Assur. Fluoresc. Meas. I Tech..

[37] Salman B.I., Hassan A.I., Al-Harrasi A., Ibrahim A.E., Saraya R.E. (2024). Copper and nitrogen-doped carbon quantum dots as green nano-probes for fluorimetric determination of delafloxacin; characterization and applications. Anal. Chim. Acta.

[38] Salman B.I., Batakoushy H.A., Sarya R.E., Hassan A.I., Al-Harrasi A., Ibrahim A.E. (2025). Comprehensive Investigation of Prunus armeniaca for Natural Green Synthesis of Carbon Quantum Dots; Applications as Fluorescent Nano-probes for Ramipril. Talanta.

[39] Tanwar A.S., Chanu M.A., Parui R., Barman D., Im Y.-H., Krishnan Iyer P. (2024). Dynamic quenching mechanism based optical detection of carcinogenic Cr(vi) in water and on economical paper test strips via a conjugated polymer. RSC Appl. Polym..

[40] Xiao Y., Xiao J., Zhao H., Li J., Zhang G., Zhang D., Guo X., Gao H., Wang Y., Chen J. (2024). Prussian Blue Analogues for Sodium-Ion Battery Cathodes: A Review of Mechanistic Insights, Current Challenges, and Future Pathways. Small.

[41] Rashdan H.R.M., Batakoushy H.A., Magdy G., Morsy M., Elzwawy A. (2024). Feasible synthesis and physicochemical features of a luminescent cadmium-metal organic frameworks (Cd-MOFs) composite, and its functionalization as a turn-off sensor towards selective determination of bisphenol A in food, water, and paper products. Microchem. J..

[42] Kumar Panigrahi S., Kumar Mishra A. (2019). Inner filter effect in fluorescence spectroscopy: As a problem and as a solution. J. Photochem. Photobiol. C Photochem. Rev..

[43] Branch S.K. (2005). Guidelines from the international conference on harmonisation (ICH). J. Pharm. Biomed. Anal..

[44] Oymak T., Dural E. (2022). Determination of sunset yellow, allura red, and fast green using a novel magnetic nanoadsorbent modified with Elaeagnus angustifolia based on magnetic solid-phase extraction by HPLC. Braz. J. Pharm. Sci..

[45] Hosseini S.F., Heidari T., Zendegi-Shiraz A., Ameri M. (2025). Application of chemometrics based on digital image analysis for simultaneous determination of tartrazine and sunset yellow in food samples. Food Chem..

[46] Xu J., Sun J., Yan F., Zhang H., Ma R., Zang Y., Guan S., Wang X. (2022). Fluorescence sensing performance of carbon dots of functionalization toward sunset yellow. Part. Part. Syst. Charact..

[47] Nowak P.M., Kościelniak P. (2019). What color is your method? Adaptation of the RGB additive color model to analytical method evaluation. Anal. Chem..

[48] Pena-Pereira F., Wojnowski W., Tobiszewski M. (2020). AGREE—Analytical GREEnness Metric Approach and Software. Anal. Chem..

[49] Wojnowski W., Tobiszewski M., Pena-Pereira F., Psillakis E. (2022). AGREEprep–analytical greenness metric for sample preparation. TrAC Trends Anal. Chem..

[50] Mansour F.R., Płotka-Wasylka J., Locatelli M. (2024). Modified GAPI (MoGAPI) tool and software for the assessment of method greenness: Case studies and applications. Analytica.

[51] Ibrahim A.E., Alamir S.G., Maged K., Magdy G., Salman B.I., Al-Harrasi A. (2024). Leveraging Green Chromatography in Quality-by-Design-Driven Study for the Simultaneous Analysis of Essential Single-Pill Antidiabetic Drug Combinations. J. Sep. Sci..

