# Supplementary material for: Smart Prussian Blue Analog Decorated with Zinc Oxide Nanohybrid: Fluorescent Sensing and Sustainability of Sunset Yellow in Food and Environment
Source: Biosensors (Basel). 2025 Apr 20;15(4):263. doi: 10.3390/bios15040263 (PMC12025978; doi:10.3390/bios15040263)
Supplement: Supplementary file 1 [file biosensors-15-00263-s001.zip › biosensors-3519915-supplementary.pdf]

Supplementary Materials

Smart Prussian Blue Analog Decorated with Zinc Oxide Nanohybrid: Fluorescent Sensing and Sustainability of Sunset Yellow in Food and Environment

Hany A. Batakoushy [1,2,*], Amr K. A. Bass [2,3], Hassanien Gomaa [4], Sami El Deeb [5,*] and Adel Ehab Ibrahim [6,7,*]

[1] Department of Pharmaceutical Analytical Chemistry, Faculty of Pharmacy, Menoufia University, Shebin Elkom 32511, Egypt
[2] Department of Pharmaceutical Analytical Chemistry, Faculty of Pharmacy, Menoufia National University, 70 km Cairo-Alexandria Agricultural Road, Menoufia 32952, Egypt; amrk.a.bass@phrm.menofia.edu.eg
[3] Department of Pharmaceutical Chemistry, Faculty of Pharmacy, Menoufia University, Shebin Elkom 32511, Egypt
[4] Department of Chemistry, Faculty of Science, Al-Azhar University, Assiut 71524, Egypt; h.gomaa@azhar.edu.eg
[5] Institute of Medicinal and Pharmaceutical Chemistry, Technische Universitaet Braunschweig, 38106 Braunschweig, Germany
[6] Natural and Medical Sciences Research Center, University of Nizwa, P.O. Box 33, Birkat Al Mauz, Nizwa 616, Oman
[7] Pharmaceutical Analytical Chemistry Department, Faculty of Pharmacy, Port Said University, Port Said 42511, Egypt
* Correspondence: hany.batakoushy@phrm.menofia.edu.eg (H.A.B.); s.eldeeb@tu-bs.de (S.E.D.); adel@unizwa.edu.om (A.E.I.)

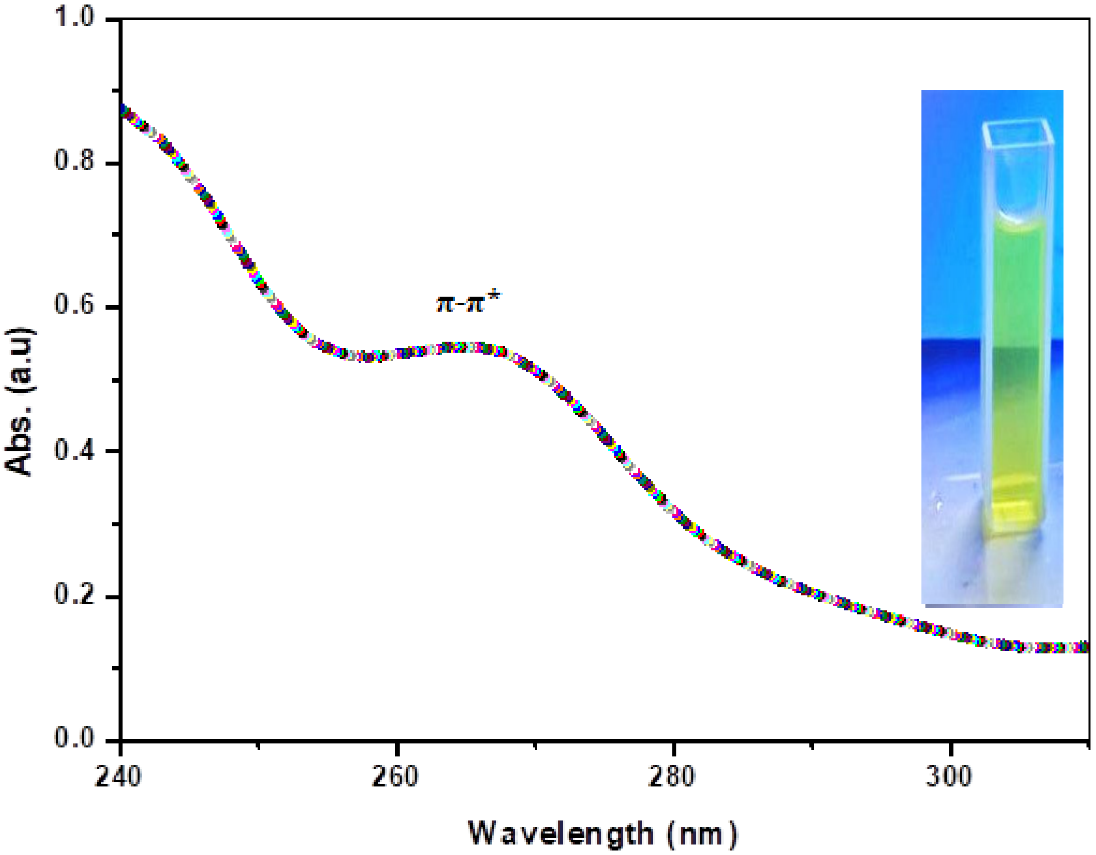

Figure S1: UV spectrum of PBA@ZnO nanohybrid (5.0 mg/mL) showing peak maximum at 270 nm.

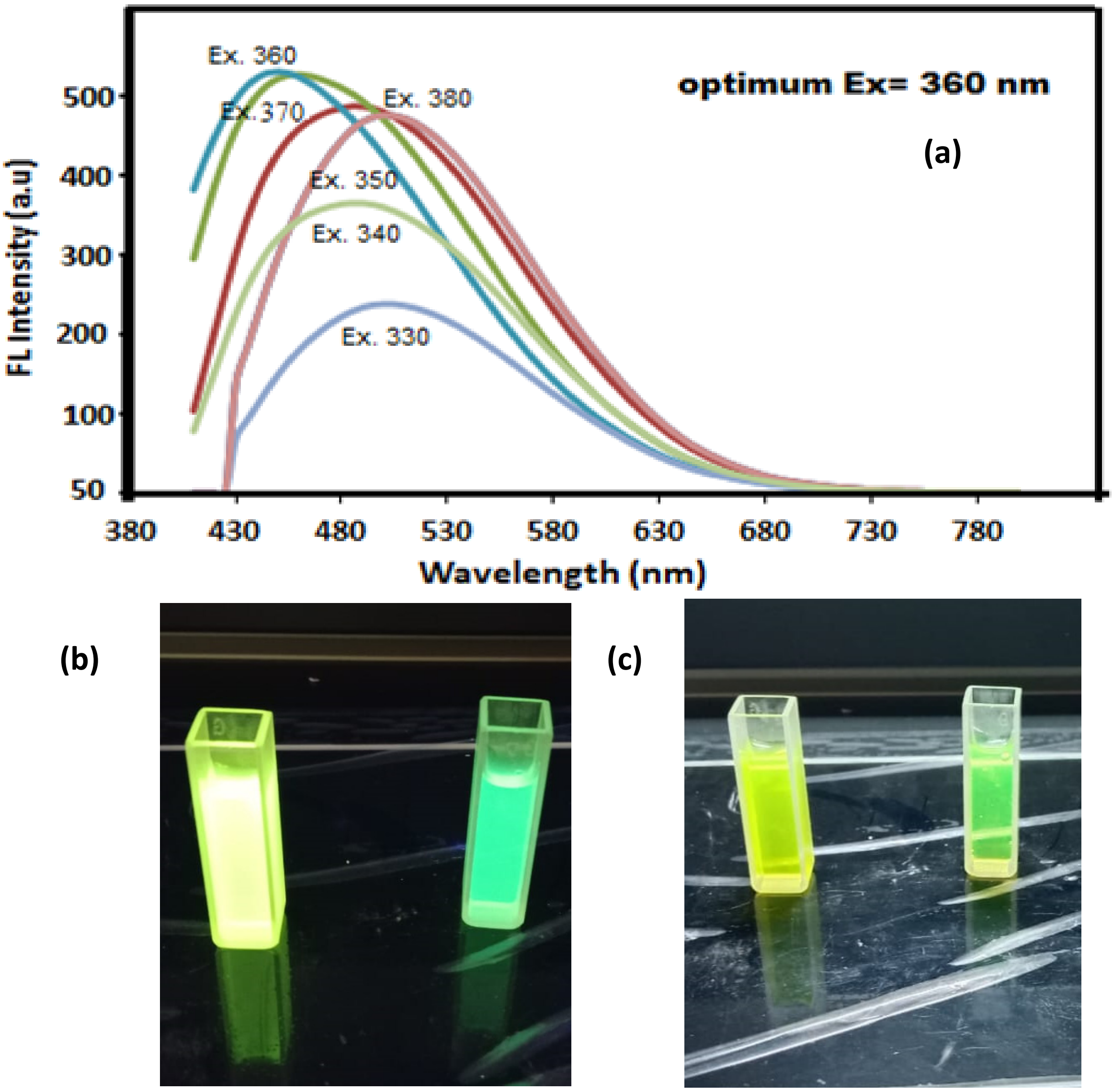

Figure S2: Fluorescence emission spectra of PBA@ZnO (5.0 mg/mL) nanohybrid at different excitation wavelengths (330-380 nm), and digital images of non-spiked PBA@ZnO under UV and white light (b&c left cuvette) and PBA@ZnO spiked with SY under UV and white light (b&c right cuvette), respectively.

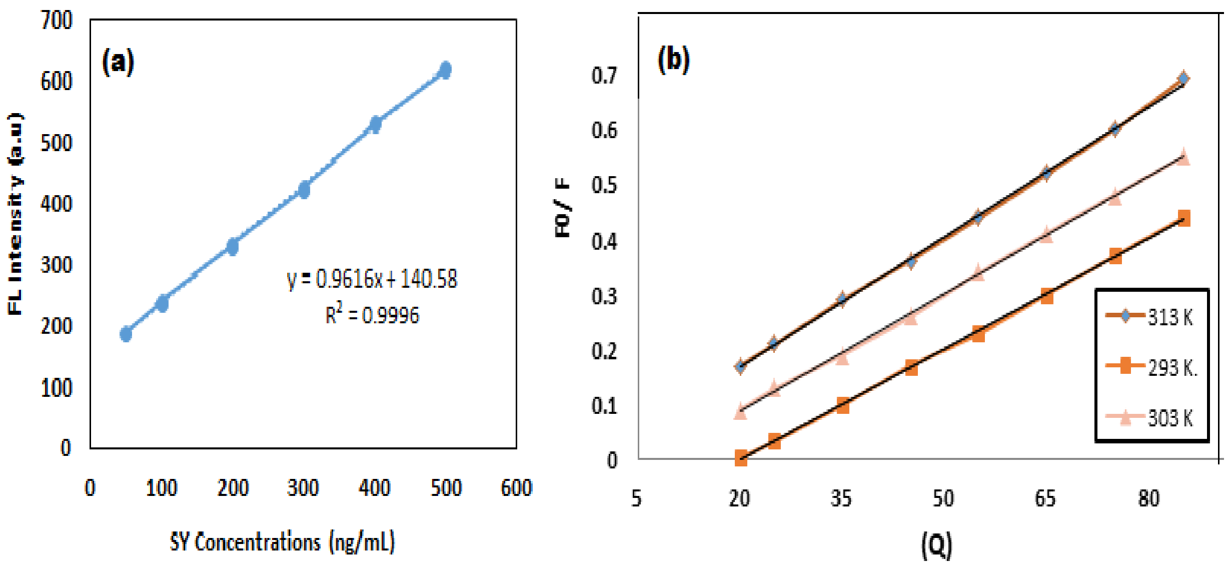

Figure S3: (a) Calibration curve of increasing concentrations of SY (0, 50, 100, 200, 300, 400, 500 ng/mL) using 0.5 mL of PBA@ZnO nanohybrid at pH 3, 1.5 mL of BR buffer, and the complete response was determined after 5 minutes, and (b) Stern-Volmer plot for the quenching effect of PBA@ZnO nanohybrid by SY at different temperatures (293, 303, 313 K).

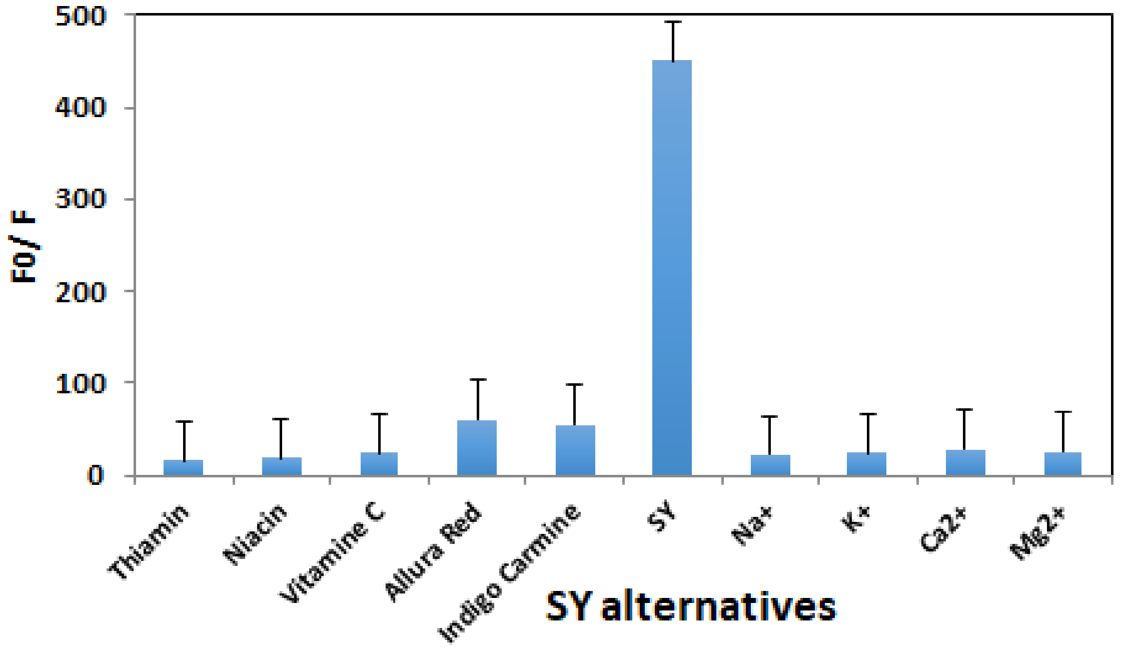

Figure S4: The selectivity of the proposed PBA@ZnO nanohybrid (100 ng/mL) with SY alternatives (1.0 µg/mL).

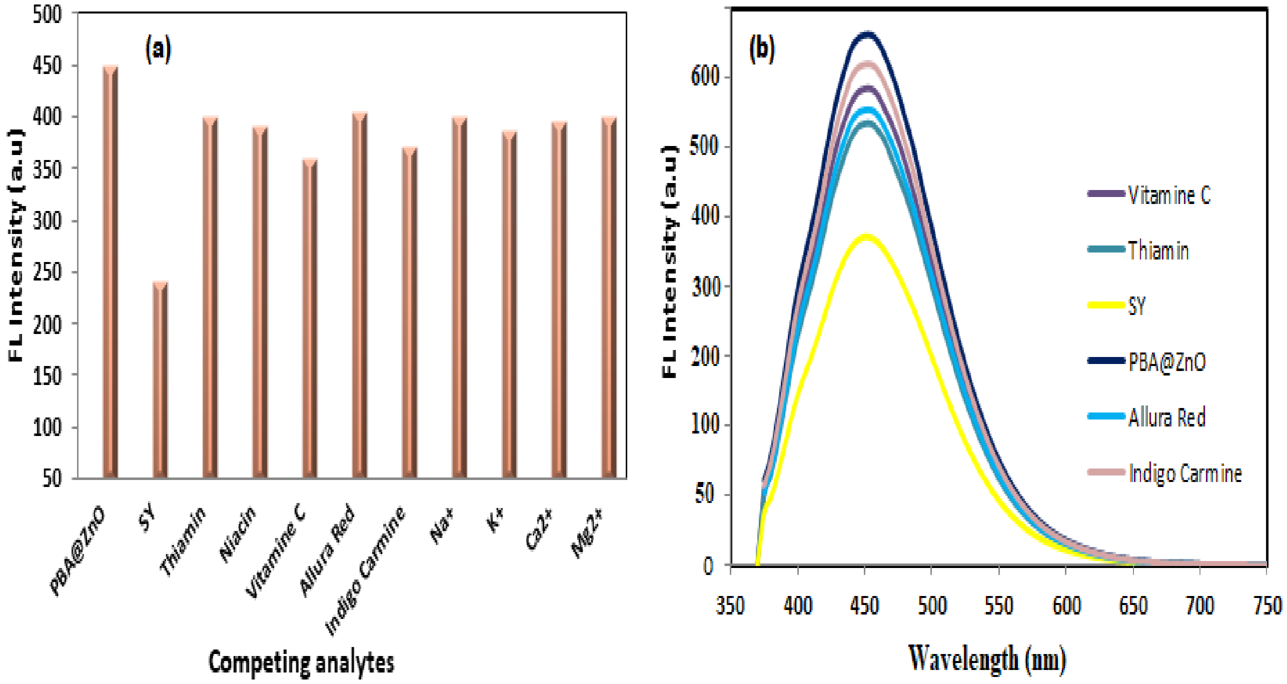

Figure S5: (a) the selectivity of the proposed PBA@ZnO nanohybrid (100 ng/mL) with competing analytes, (b) fluorescence titration with other analytes

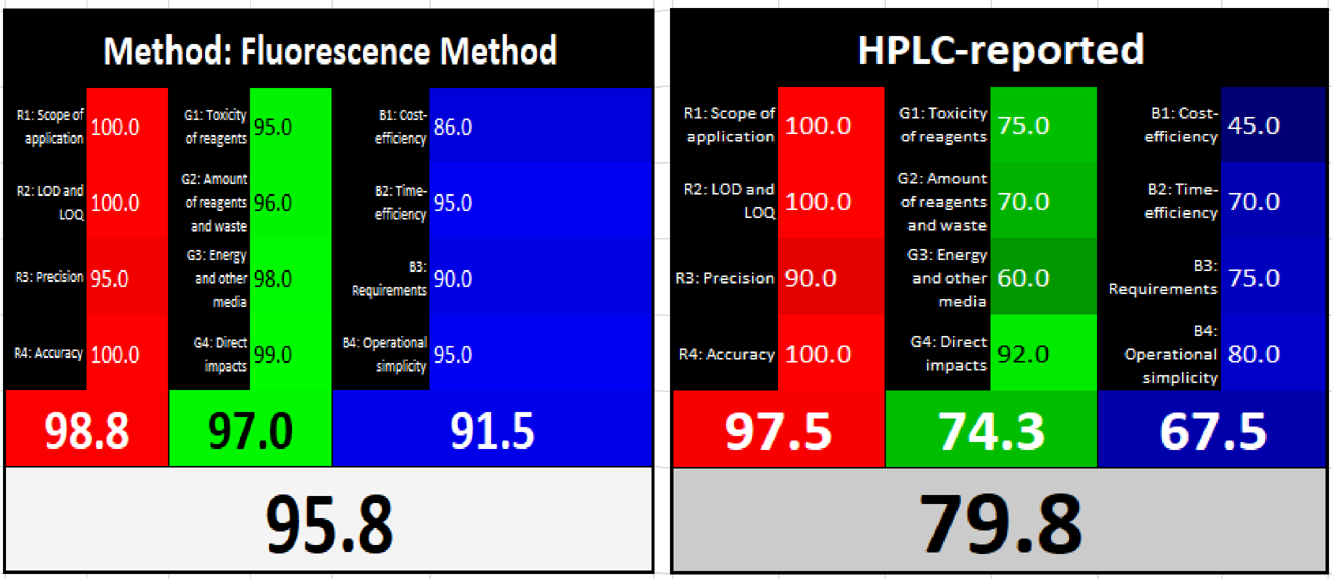

Figure S6: Comparison of RGB 12 algorithm of the suggested approach to the reported HPLC method [44].

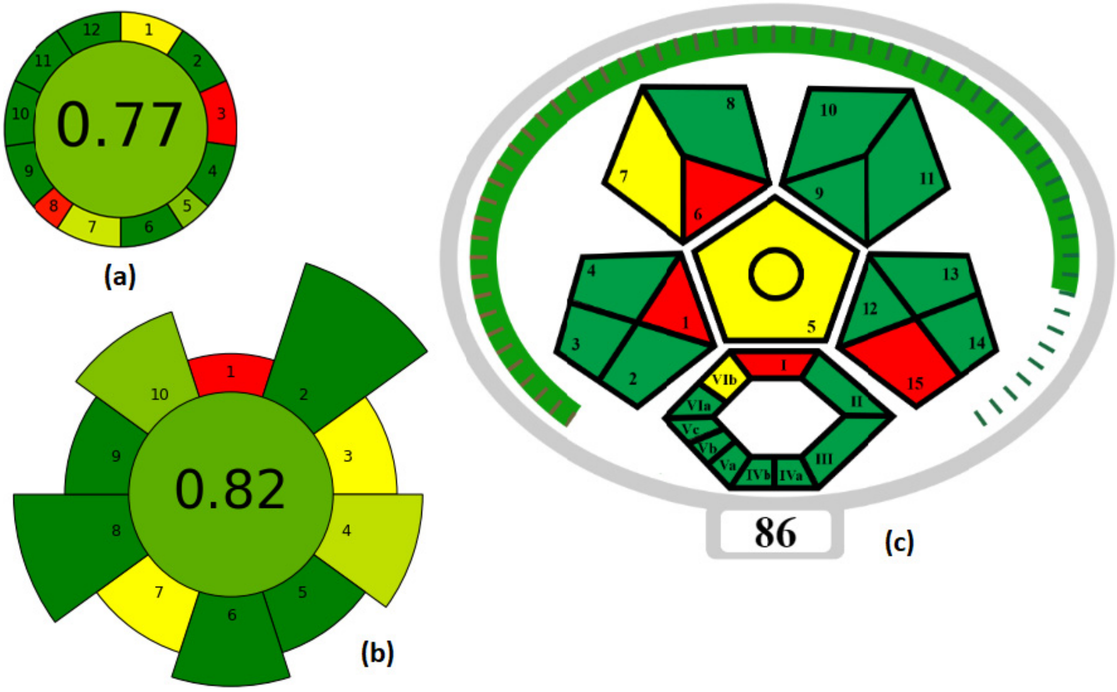

Figure S7: Assessment of the greenness profile of the proposed method by (a) AGREE, (b) AGREE prep, and (c) Complex MoGAPI.
